# Associations between liking for fat, sweet or salt and obesity risk in French adults: a prospective cohort study

**DOI:** 10.1186/s12966-016-0406-6

**Published:** 2016-07-04

**Authors:** Aurélie Lampuré, Katia Castetbon, Amélie Deglaire, Pascal Schlich, Sandrine Péneau, Serge Hercberg, Caroline Méjean

**Affiliations:** Equipe de Recherche en Epidémiologie Nutritionnelle, Centre de Recherche en Epidémiologie et Statistiques, Inserm (U1153), Inra (U1125), Cnam, COMUE Sorbonne Paris Cité, Université Paris 13, 74 rue Marcel Cachin, F-93017 Bobigny Cedex, France; Institut de veille sanitaire (InVS), Unité de surveillance et d’épidémiologie nutritionnelle (USEN), Université Paris 13, F-93017 Bobigny, France; Ecole de Santé Publique, Centre de Recherche en Epidémiologie, Biostatistiques et Recherche Clinique, Université Libre de Bruxelles, B-1070 Bruxelles, Belgique; Inra, UMR 1253, Science et Technologie du Lait et de l’Œuf, Rennes, France; Centre des Sciences du Goût et de l’Alimentation, UMR 6265 CNRS, UMR 1324 Inra, Dijon, France; Département de Santé Publique, Hôpital Avicenne (AP-HP), Bobigny, F-93017 France

**Keywords:** Obesity, Fat sensation, Salty taste, Sweet taste, Sensory liking, Dietary intake, Mediating factor

## Abstract

**Background:**

Individual sensory liking appears to be an important determinant of dietary intake and may consequently influence weight status. Cross-sectional studies have shown positive association between fat liking and weight status and equivocal results regarding salt and sweet liking. Moreover, the contribution of dietary intake to explain this relationship has not been studied yet. We investigated the prospective association between sensory liking for fat, sweet or salt and the onset of obesity over 5 years in adults, and the mediating effect of dietary intake.

**Methods:**

We prospectively examine the risk of obesity among 24,776 French adults participating in the NutriNet-Santé cohort study. Liking scores and dietary data were assessed at baseline using a validated web-based questionnaire and 24 h records, respectively. Self-reported anthropometric data were collected using web-based questionnaire, each year during 5 years. Associations between quartiles of liking for fat, sweet or salt and obesity risk, and the mediating effect of diet were assessed by multivariate Cox proportional hazards models stratified by gender, adjusted for sociodemographic and lifestyle factors.

**Results:**

In both genders, sensory liking for fat was associated with an increased risk of obesity (hazard ratios for quartile 4 compared to quartile 1, men: HR_Q4vs.Q1_ = 2.39 (95 % CI 1.39,4.11) P-trend = 0.0005, women: HR_Q4vs.Q1_ = 2.02 (1.51,2.71) P-trend = <0.0001). Dietary intake explained 32 % in men and 52 % in women of the overall variation of liking for fat in obesity. Sensory liking for sweet was associated with a decreased risk of obesity (men: HR_Q4vs.Q1_ = 0.51 (0.31,0.83) P-trend = 0.01, women: HR_Q4vs.Q1_ = 0.72 (0.54,0.96) P-trend = 0.035). No significant association between salt liking and the risk of obesity was found.

**Conclusions:**

Unlike sweet and salt liking, higher liking for fat appears to be a major risk factor of obesity, largely explained by dietary intake. Our findings emphasize the need to centrally position sensory liking in obesity prevention.

## Background

A large body of literature has suggested the possible role of excessive consumption of fat, sugar and sodium in the etiology of major chronic diseases, including obesity, cardiovascular diseases and some cancers [1, 2]. Most public health programs worldwide target nutritional recommendations, which include limitations on fat, salt and sugar in dietary intake [2]. However, these components contribute to eating pleasure due to the sensory properties they drive, which may promote their overconsumption [3]. Individual sensory liking for fat, sweet or salt therefore appears to be a major determinant of dietary intake and may consequently influence weight status [4, 5].

The majority of cross-sectional studies have highlighted a positive association between fat liking and body mass index (BMI) [5–14] while three studies only have shown no significant association [15–17]. Such discrepancies between studies may be explained by difference in fat liking assessment and statistical methods. Findings regarding sweet and salt liking are more equivocal. Experimental studies have reported no difference in sweet liking across BMI or even a lower sweet liking in obese [7, 15, 18, 19] and one study have reported high sweet liking in lean participants but not in obese individuals [20]. The observational studies conducted in general population found a positive association between sweet liking and BMI [5, 6]. Sweet liking seemed to varied across stimuli [6, 8] and distinct types of hedonic response has been identified regarding liking for sweet [18] which can explained such various findings. Finally, some studies have shown no association between salt liking and BMI [20–23] and other works have highlighted direct [5, 16, 24] or inverse relationships [25]. A recent study also showed that obese were more sensitive to salty taste than non-obese individuals [26]. As cross-sectional design does not allow causal inferences, longitudinal studies assessing the influence of sensory liking on obesity are therefore needed. Only two prospective studies have been conducted regarding the influence of liking for fat and for sweet on change in weight status over time [27, 28]. Salbe et al. have highlighted that a heightened response for sweet and creamy solutions was associated with 5-year weight gain in Pima Indians [28]. However, this study has been conducted in small and highly selected populations, which does not allow generalizing results. Another prospective study has shown that Japanese subjects who liked sweet taste experienced a significantly increase of weight during the 10 years of follow-up and no such difference was found for “rich and heavy” taste, equivalent to fat sensation [27]. Nevertheless, the latter study assessed liking only through two questions, which is not a reliable measure of overall liking [29].

To our knowledge, no study has investigated the contribution of dietary intake to explain the influence of sensory liking on weight status. Even if dietary fat intake is not directly associated with weight change [30], the influence of fat liking on weight status may therefore be mediated by overall dietary intake rather than fat intake. Previous studies have shown that subjects with high fat liking have higher fat intake but also lower intake of nutrient-dense foods such as fruits and vegetables, dairy products, whole grains products and fish, compared to those with low liking [4, 31–33] which may increase the risk of weight gain and obesity [2]. This emphasizes the need to consider the overall dietary intake, particularly intake of nutrient-dense foods, and not only specific nutrients intake in the relationship between high liking for fat, sweet or salt and the risk of obesity.

The aim of our study was to assess the prospective association between individual liking for fat, sweet or salt and the risk of developing obesity over 5 years, in a large population of French adults. In addition, we also investigated the mediating effect of dietary intake of energy and food groups on the relationship between sensory liking and obesity.

## Methods

### Study population

We used data from the NutriNet-Santé study [34] (in English the NutriNet-Health study), a large web-based observational cohort launched in France in 2009 with a scheduled follow-up of 10 years. It was implemented in a general population and targeted Internet-using adult volunteers. Eligible participants were recruited by a vast multimedia campaign (television, radio, national and regional newspapers, posters, and Internet) called for volunteers and provided details about the study’s website [35]. Recruitment information is now maintained on a large number of websites and is regularly updated via professional channels. The key message delivered in the call for volunteers was that nutrition is a protective factor of chronic diseases and in order to highlight specific role of nutritional factors, development of cohort studies is essential. The purpose of the study is to identify nutritional risk factors or protective factors for chronic diseases, which is an essential step in establishing dietary recommendations to prevent the risk of disease and improve the health of the current and future generations. Aspects related to convenience of participation (i.e., ≤20 min each month) and confidentiality were also emphasized [36]. In addition, a system of boosting motivation and retention was implemented.

Briefly, in order to be included in the cohort, participants had to fill out an initial set of questionnaires assessing dietary intake, physical activity, anthropometry, lifestyle, socio-economic conditions and health status. As part of their follow-up, the participants complete the same set of questionnaires every year. Moreover, each month, they are invited to fill out complementary questionnaires related to determinants of food behavior, nutritional and health status. All questionnaires were completed online via the NutriNet-Santé website.

### Data collection

#### Assessment of liking for fat, sweet and salt

Liking for fat, sweet and salt was assessed using PrefQuest, an original web-based questionnaire [29]. This questionnaire assesses overall liking for fat, saltiness and sweetness via several items, enabling an assessment of overall liking, i.e. liking primarily derived from sensation independently of the food product. It was internally validated by studying the underlying structure of each taste using exploratory factor analysis and confirmatory factor analysis [29], and also compared with sensory tests that included 32 food models conducted in a diversified sample (*n* = 557) [37] (Deglaire et al. 2011, personal communication). The salty taste was unidimensional, unlike the sweet taste and the fat sensation. The sweet taste was formed by the factors ‘sweet foods’, ‘added sugar’ and ‘natural sweetness’ and the fat sensation was composed of the fat-and-salt sensation based on ‘added fat-and-salt’ and ‘fatty-salty foods’ and the fat-and-sweet sensation based on ‘added fat-and-sweet’ and ‘fatty-sweet foods’ [29].

Briefly, PrefQuest is composed by 83 relevant items divided into liking for salt (11 items) and sweet (21 items) tastes, fat-and-salt (31 items) and fat-and-sweet (20 items) sensations. The questionnaire included four types of items: (i) liking for sweets, fatty-sweet and fatty-salty foods, rated on a 9-point hedonic scale from “I don’t like it/them at all” to “I like it/them very much” (example: How much do you like hamburgers?); (ii) preferred level of salt, sweet, fat-and-salt or fat-and-sweet seasoning using pictures, measured on a 6-point scale from “with no” to “with a lot of” (example: How do you prefer your steak?); (iii) preferred drinks (sweet/sweetened or unsweetened) on a restaurant menu as a multiple choice question; and (iv) dietary behavior in terms of sweet, salty and fatty foods, measured on a 5-point frequency scale from “never” to “always” (example: Do you salt your dish before tasting?) or a 9-point scale from “not at all” to “a lot” (example: Do you ever eat jam straight out of the jar with a spoon?). For most items, subjects also had the option of checking a non-applicable answer, such as “I have never tasted [this food]” or “I do not like [this food]”. In May 2010, 65,683 participants of the NutriNet-Santé cohort were invited to complete this optional questionnaire.

#### Anthropometric measurements

Height and weight data were collected at enrollment and each year thereafter by a self-administered anthropometric questionnaire [38]. BMI (kg/m^2^) was calculated as the ratio of weight to the square of height. Participants with BMI < 25 were classified as underweight/normal weight, participants with 25 ≤ BMI < 30 were considered overweight (excluding obese) and participants with BMI ≥ 30 were considered obese in accordance with WHO reference values [39]. The closest available anthropometric data to the PrefQuest questionnaire were used as the baseline data.

#### Assessment of dietary intake

At enrollment and each year thereafter, participants were invited to provide three random 24 h dietary records during a two-week period (1 weekend day and 2 weekdays) [38]. The web-based tool is designed for self-administration on the Internet and based on a secured user-friendly interface, designed by Medical Expert Systems MXS. The web-based dietary assessment method relies on a meal-based approach, recording all foods and beverages (type and quantity) consumed at breakfast, lunch, dinner and all other eating occasions. First, the participant fills in the names of all food items eaten. Next, he/she estimates portion sizes for each reported food and beverage item according to standard measurements (e.g. home containers, grams indicated on the package) or using images available via the interactive interface. These photographs, taken from a validated picture booklet [40], represent more than 250 foods (corresponding to 1 000 generic foods) served in seven different portion sizes. The values for energy were estimated using published nutrient databases [41] and completed for recent market foods and recipes. The accuracy of web-based 24 h dietary records has been assessed by comparing to interviews by trained dietitians [38] and also against 24 h urinary biomarkers [42, 43]. Foods were classified according to the information provided in the French National Nutrition and Health Program guides [44]. Food groups (in grams/day) considered in the present study were vegetables, fruits, meat, processed meat, fish, starchy foods, whole grain products, cheese, milk and yogurt, sugar and sweetened products, sweetened cream desserts, fatty-sweet products, savory sauces, salted snacks and appetizers, oils, butter and other added fats, sugar-sweetened soft drinks, artificially sweetened drinks and alcoholic beverages.

#### Sociodemographic, lifestyle and behavioral data

Potential confounding factors of the relationship between sensory liking for fat, sweet or salt and the risk of obesity previously identified [5, 45, 46] were collected using web-based questionnaires at the same time as sensory liking data: age (years), sex, education (elementary school, secondary school, college graduate or advanced degree), smoking status (never, former or current smoker), alcohol consumption (abstainer and irregular consumer, moderate consumer or heavy drinker), history of dieting (never, former, or current dieter) and physical activity level using the French version of the International Physical Activity Questionnaire [47] (low, moderate or high).

#### Statistical analyses

The present analysis focused on participants of the NutriNet-Santé cohort, living in metropolitan France, who had completed the PrefQuest and the set of questionnaires, and who had self-reported weight and height data over 5 years of follow-up. All analyses were performed separately for men and women, since significant sex interactions were found (*P* < 0.05).

Liking scores for fat, sweet and salt were computed as detailed previously [29, 45]. Briefly, all data were linearly transformed into values ranging from 0 to 10 to standardize ratings. First, for each participant, liking scores of each factor composing a sensation/taste were calculated by summing the ratings of the items belonging to the factor and dividing by the number of items of this factor. Next, overall liking scores of a sensation/taste were computed by averaging liking scores of compounding factors. Then, quartiles of scores for fat, sweet and salt were computed in order to define liking levels, from quartile 1 “lowest liking” to quartile 4 “highest liking”.

Regarding dietary intake, for each participant, daily mean quantities of the food group (in grams) and energy intake were calculated from at least two 24 h records, weighted according to the day (week or weekend) with the closest data to the PrefQuest questionnaire. Diet-underreporting participants were identified by the method proposed by Black [48]. Briefly, basal metabolic rate (BMR) was estimated by Schofield equations [49] according to sex, age, weight and height collected at enrollment in the study. Energy intake and BMR were compared to a physical activity level of 1.55 or below, the WHO value for ‘light’ activity, to identify energy-underreporting subjects [48]. They were consequently excluded for analysis. Subjects with two 24 h dietary records (*n* = 1141, 4.6 % of the sample), compared to those with three 24 h records, had slightly higher liking for fat and sweet and similar liking for salt. Finally, regarding weight, typing error were identified as aberrant data and deleted.

Comparisons between included and excluded participants were performed using Student’s *t*-test and chi-square test, as appropriate. Mean liking scores for fat, sweet and salt were compared between men and women, and between obese participants (baseline or follow-up) using Student’s *t*-test, and were presented with their standard deviation. Individual characteristics and dietary intake according to quartiles of liking for fat, sweet and salt were compared using analysis of covariance and chi-square test, as appropriate. Comparisons between obese and non-obese participants were performed using Student’s *t*-test and chi-square test, as appropriate. Cox proportional hazard models, stratified for sex and with age as the primary time variable, were used to calculate hazard ratios (HR) and 95 % confidence intervals for the association between quartiles of liking for fat, sweet and salt and the risk of obesity (1^st^ quartile of liking level as reference). First, Cox base models were performed to study the independent effect of liking for fat, sweet and salt on the risk of developing obesity. Then, to assess the mediating effect of dietary intake, we selected food groups for which the intake was associated with obesity risk as well as liking for fat, sweet or salt using Cox or logistic regression models, as appropriate (P ≤ 0.1) [50]. Secondly, Cox models assessing the mediating effect of dietary intake on the relationship between sensory liking and the risk of obesity were performed adjusted for daily energy intake and month of inclusion to take into account the seasonality in dietary data collection. Thirdly, fully adjusted Cox models were performed by adding confounding factors previously mentioned.

The magnitude of the mediating effect was assessed by the percentage change in the HRs of the different liking groups computed as [(HR base model – HR base model + mediator)/(HR base model − 1)] × 100 [51]. Dietary intake was considered as a mediating factor when the percentage change of the HR in at least one of the liking level groups was higher than 10 % and there was no increase of other HRs. Furthermore, we calculated the part of the reduction in deviance attributable to sensory liking for fat sensation, sweet or salty taste, which was accounted for by inclusion of the potential mediator and confounders in the model. This reduction in deviance related to sensory liking, used as an overall statistical test of the mediating effect, quantifies the percentage of the sensory liking impact on the outcome explained by the mediator [52]. The deviance of the model is the mathematical function which compares the observed values of the response variable to those predicted by the model. The deviance of fat, sweet and salt liking in the base model was compared to the deviance of fat, sweet and salt liking in the extended model and the percentage of reduction of deviance due to sensory liking (RD) explained by inclusion of the mediating factor or confounders was calculated as follows [(RD due to sensory liking in base model) − (RD due to sensory liking in base model + mediator/confounders)/RD due to sensory liking in base model] × 100 [51]. To optimize the robustness of the statistical tests, we performed sensitivity analyses. First, we reanalyzed our data after including participants with missing data for confounding factors. Second, we redefined the outcome as the risk of becoming overweight in order to overcome the potential misclassification bias.

The actuarial method was used and assumptions of proportionality were satisfied through examination of the log-log (survival) compared with log-time plots. Data management and statistical analyses were performed using SAS software (version 9.3, SAS Institute Inc, Cary, NC, USA). Tests for linear trend were performed using the ordinal score on quartiles of liking for fat, sweet and salt. A *P*-value <0.05 was considered statistically significant.

## Results

Among the 65,683 participants in the NutriNet-Santé study in May 2010, 49,066 responded to the PrefQuest (75 % participation rate). Among responders, 39,540 had available data for height and weight in 2010. Then, we excluded 4137 subjects who were obese at baseline, 55 participants with aberrant data for height and weight, 4732 lost of follow-up as well as 1528 women who were pregnant at specific time during the follow-up. Moreover, 4312 subjects with missing data for physical activity, alcohol consumption and history of dieting were excluded, which left 24,776 participants available for analysis (18,601 women and 6175 men). Compared with excluded subjects, individuals included in our analysis were older, the percentages of men, of those with high education and of those with no history of dieting were higher, and the proportion of smokers was lower (data not shown).

During a median follow-up of 4 y, 664 individuals became obese (503 women and 161 men). In women (Table 1), fat liking score ranged from 0 to 8.8 with a mean of 3.8 ± 1.4, sweet liking score ranged from 0 to 9.4 with a mean of 3.6 ± 1.3 and salt liking score ranged from 0 to 10 with a mean of 3.8 ± 1.5. In men (Table 1), fat liking score ranged from 0 to 9.6 with a mean of 4.0 ± 1.4, sweet liking score ranged from 0 to 9.6 with a mean of 4.0 ± 1.4 and salt liking score ranged from 0 to 10 with a mean of 3.8 ± 1.6. Furthermore, an additional analysis showed no significant difference in mean liking scores for fat, sweet and salt between subjects obese at baseline (who were excluded of the analysis sample) and subjects who became obese during the follow-up (data not shown).

**Table 1:** Quartiles of liking scores for the fat sensation, sweet and salty tastes, *n* = 24,776, NutriNet-Santé cohort, France

Liking scores	Sex	Mean	SD ^a^	Quartile 1 “Low liking”	Quartile 2	Quartile 3	Quartile 4 “High liking”
Fat	Women	3.75 ^b^	1.40	0.00, 2.76	2.76, 3.65	3.65, 4.64	4.64, 8.80
	Men	3.95	1.42	0.09, 2.97	2.97, 3.86	3.86, 4.84	4.84, 9.61
Sweet	Women	3.64 ^b^	1.28	0.00, 2.72	2.72, 3.54	3.54, 4.47	4.47, 9.35
	Men	4.03	1.35	0.12, 3.07	3.07, 3.97	3.97, 4.94	4.94, 9.55
Salt	Women	3.76	1.52	0.00, 2.77	2.77, 3.77	3.77, 4.72	4.72, 10.0
	Men	3.80	1.59	0.00, 2.76	2.77, 3.82	3.82, 4.86	4.87, 10.0

^a^ Standard deviations

^b^ Different from men, *P* < 0.0001

Sociodemographic characteristics, lifestyle and dietary intake across quartiles of liking scores for fat, sweet and salt stratified by sex are presented in Tables 2 and 3. Men and women with higher liking for fat, sweet or salt (quartile 4) were more often current smokers, were less physical active, and had higher intake of energy, meat, processed meat and sugar-sweetened soft drinks, whereas they had lower intake of fruits and whole grain products compared with participants with lower liking scores. Furthermore, subjects with high fat liking and high sweet liking where younger, whereas those with high salt liking were older and were more often heavy drinkers.

**Table 2:** Baseline characteristics in men *n* = 6175, NutriNet-Santé cohort, France

	Quartiles of liking for fat					Quartiles of liking for sweet					Quartiles of liking for salt				
	Q1	Q2	Q3	Q4	*P* *	Q1	Q2	Q3	Q4	*P*	Q1	Q2	Q3	Q4	*P*
General characteristics															
Age, y	59.4 ± 11.4 ^a^	54.3 ± 13.8	51.0 ± 14.6	45.6 ± 15.0	<0.0001	56.0 ± 12.8	53.2 ± 14.1	51.6 ± 14.9	49.6 ± 15.9	<0.0001	50.4 ± 14.6	51.7 ± 14.6	53.4 ± 14.8	54.9 ± 14.2	<0.0001
BMI, kg/m^2^	24.2 ± 2.6	24.2 ± 2.5	24.3 ± 2.6	24.1 ± 2.7	0.22	24.3 ± 2.5	24.2 ± 2.6	24.2 ± 2.6	24.0 ± 2.6	0.02	23.8 ± 2.6	24.0 ± 2.6	24.3 ± 2.6	24.6 ± 2.6	<0.0001
Educational level, %					0.002					0.99					0.21
Elementary school	4.2	2.8	3.0	2.7		3.3	3.1	3.0	3.2		3.5	2.4	2.8	4.0	
Secondary school	37.0	31.4	31.1	32.8		33.6	31.8	32.9	34.1		31.0	32.8	33.6	35.1	
College graduate	22.1	23.0	25.1	23.5		23.4	23.8	23.2	23.2		23.8	24.2	22.8	22.8	
Advanced degree	36.0	42.0	40.0	40.6		38.9	40.7	40.2	38.9		40.9	40.0	40.2	37.5	
Other	0.7	0.8	0.8	0.4		0.8	0.6	0.7	0.6		0.8	0.6	0.6	0.6	
Alcohol consumption^b^, %					0.18					0.001					<0.0001
Abstainer and irregular consumer	30.6	26.9	28.8	29.3		28.3	26.9	27.0	33.4		38.2	29.7	24.8	22.8	
Moderate consumer	56.1	58.5	57.8	55.3		56.6	58.8	58.6	53.6		53.5	57.7	61.2	55.3	
Heavy drinker	13.3	14.6	13.4	15.4		15.1	14.3	14.4	13.0		8.3	12.6	14.0	21.9	
Smoking status, %					<0.0001					<0.0001					<0.0001
Never-smoker	37.1	40.9	43.5	48.4		36.4	41.1	43.4	49.0		50.9	42.8	42.6	33.5	
Former smoker	55.0	47.2	41.3	33.5		53.0	45.3	41.5	37.3		38.1	45.3	45.3	48.4	
Current smoker	7.9	11.9	15.2	18.1		10.6	13.6	15.1	13.7		11.0	11.9	12.1	18.1	
Dieting to lose weight, %					0.38					<0.0001					0.59
Never dieter	32.1	33.0	31.9	32.3		28.0	30.9	34.4	36.1		32.4	33.7	31.6	31.6	
Former dieter	61.5	61.1	63.6	62.1		64.3	63.7	61.2	59.0		62.7	60.3	62.8	62.4	
Current dieter	6.4	5.9	4.5	5.6		7.7	5.4	4.4	4.9		4.9	6.0	5.6	6.0	
Physical activity, %					<0.0001					<0.0001					0.003
Low	14.6	20.2	21.5	25.5		15.0	20.2	22.0	24.7		17.5	19.7	20.8	23.9	
Moderate	33.8	36.0	39.5	38.1		38.4	37.0	36.8	35.2		38.5	36.8	36.8	35.3	
High	51.6	43.8	39.0	36.4		46.6	42.8	41.2	40.1		44.0	43.5	42.4	40.8	
Energy, kcal/day	2177.7 ± 511.5	2261.0 ± 523.2	2314.1 ± 532.3	2403.1 ± 572.8	<0.0001	2170.1 ± 500.4	2260.9 ± 526.7	2331.9 ± 542.1	2393.0 ± 569.5	<0.0001	2226.1 ± 536.6	2260.2 ± 513.7	2311.8 ± 513.7	2359.2 ± 571.2	<0.0001
Food group consumption, g/day															
Fruits	364.2 ± 230.2	307.1 ± 202.9	291.6 ± 193.8	268.5 ± 207.7	<0.0001	335.9 ± 222.4	312.5 ± 214.3	305.5 ± 204.2	277.4 ± 202.4	<0.0001	324.8 ± 226.8	308.6 ± 205.9	312.3 ± 208.9	285.5 ± 204.0	<0.0001
Meat	50.6 ± 49.2	60.5 ± 52.3	60.8 ± 51.0	60.2 ± 54.7	<0.0001	54.2 ± 50.1	57.3 ± 51.3	57.4 ± 50.7	63.1 ± 55.3	<0.0001	51.1 ± 49.9	54.8 ± 48.3	59.3 ± 50.4	67.0 ± 57.7	<0.0001
Processed meat	32.0 ± 36.3	36.0 ± 37.4	40.2 ± 37.8	46.9 ± 43.8	<0.0001	35.2 ± 37.7	38.1 ± 37.9	39.2 ± 40.0	42.6 ± 41.2	<0.0001	35.3 ± 38.7	36.2 ± 36.0	39.1 ± 36.9	44.5 ± 44.6	<0.0001
Milk and yogurts	167.6 ± 164.6	168.4 ± 161.8	170.4 ± 158.3	178.7 ± 166.3	0.21	170.4 ± 169.1	160.0 ± 155.5	168.7 ± 158.0	186.0 ± 167.3	0.0001	174.7 ± 161.5	175.6 ± 163.0	177.8 ± 163.5	156.8 ± 162.4	0.001
Cheese	42.5 ± 35.4	48.0 ± 34.8	48.7 ± 36.0	50.6 ± 39.6	<0.0001	44.9 ± 35.7	46.8 ± 35.8	49.8 ± 36.9	48.1 ± 37.7	0.002	44.8 ± 35.6	47.1 ± 36.4	48.3 ± 37.7	49.6 ± 36.4	0.003
Oil	10.7 ± 11.4	10.6 ± 10.5	9.4 ± 9.4	10.0 ± 10.2	0.002	10.4 ± 10.9	10.1 ± 10.3	10.5 ± 10.5	9.7 ± 9.9	0.16	10.1 ± 10.9	9.6 ± 9.6	10.6 ± 10.7	10.4 ± 10.4	0.05
Whole grain products	55.9 ± 76.0	40.3 ± 63.1	33.6 ± 53.9	31.4 ± 53.9	<0.0001	50.9 ± 72.5	42.3 ± 61.4	36.8 ± 60.0	31.1 ± 55.8	<0.0001	47.7 ± 72.1	41.6 ± 60.7	37.2 ± 59.7	34.6 ± 58.1	<0.0001
Sugar and sugary products	29.7 ± 29.4	32.0 ± 30.7	30.1 ± 30.1	28.0 ± 29.5	0.003	20.5 ± 25.3	28.4 ± 27.3	33.3 ± 30.3	37.6 ± 33.5	<0.0001	27.6 ± 29.5	30.7 ± 30.3	30.8 ± 28.3	30.8 ± 31.5	0.005
Fatty-sweet products	52.7 ± 57.4	70.8 ± 62.7	81.2 ± 70.8	92.8 ± 76.5	<0.0001	59.4 ± 62.2	70.1 ± 65.7	81.3 ± 72.3	86.5 ± 71.6	<0.0001	78.4 ± 73.6	75.4 ± 69.0	71.1 ± 64.5	72.5 ± 67.7	0.02
Sugar-sweetened soft drinks	29.3 ± 79.9	39.1 ± 97.3	49.2 ± 132.5	75.1 ± 149.9	<0.0001	29.3 ± 80.6	37.9 ± 101.8	56.4 ± 124.3	69.1 ± 154.1	<0.0001	46.5 ± 106.4	45.6 ± 121.8	46.8 ± 109.4	54.0 ± 137.5	0.18
Artificially sweetened drinks	20.4 ± 107.0	28.4 ± 129.9	26.2 ± 114.8	37.1 ± 128.9	0.002	25.8 ± 115.2	29.1 ± 130.3	28.4 ± 116.0	28.9 ± 120.5	0.86	24.7 ± 111.2	25.5 ± 111.6	31.9 ± 126.0	30.1 ± 132.6	0.26

* *P* values are for the comparison between sex-specific quartiles of liking for fat, sweet and salt and were determined by using analysis of covariance or chi-square tests as appropriate

^a^ Mean ± SD (all such values)

^b^ Male drinkers were categorized as abstainer and irregular consumer (≤4 g alcohol/day), moderate consumer (>4 to ≤30 g alcohol/day), or heavy drinker (>30 g alcohol/day)

**Table 3:** Baseline characteristics in women *n* = 18,601, NutriNet-Santé cohort, France

	Quartiles of liking for fat					Quartiles of liking for sweet					Quartiles of liking for salt				
	Q1	Q2	Q3	Q4	*P* *	Q1	Q2	Q3	Q4	*P*	Q1	Q2	Q3	Q4	*P*
General characteristics															
Age, y	53.9 ± 11.7 ^a^	48.6 ± 13.2	44.1 ± 13.4	40.9 ± 13.0	<0.0001	51.3 ± 12.4	48.5 ± 13.0	45.6 ± 13.6	42.1 ± 14.0	<0.0001	45.4 ± 14.0	47.5 ± 13.7	47.6 ± 13.4	47.0 ± 13.6	<0.0001
BMI, kg/m^2^	22.2 ± 2.9	22.5 ± 2.9	22.5 ± 2.8	22.5 ± 2.9	<0.0001	22.4 ± 2.9	22.5 ± 2.8	22.6 ± 2.8	22.2 ± 2.8	<0.0001	22.2 ± 2.9	22.4 ± 2.9	22.5 ± 2.8	22.6 ± 2.9	<0.0001
Educational level, %					<0.0001					0.0003					0.66
Elementary school	3.1	2.5	2.2	2.0		3.1	2.5	2.2	2.0		2.7	2.7	2.3	2.3	
Secondary school	37.3	31.4	29.5	32.9		33.5	32.8	31.5	33.2		33.1	33.1	32.2	32.7	
College graduate	30.3	32.4	33.5	33.4		31.9	31.4	32.1	34.1		31.1	32.6	32.8	33.0	
Advanced degree	28.6	33.0	34.2	31.2		30.8	32.4	33.6	30.2		32.4	31.0	32.1	31.4	
Other	0.7	0.7	0.6	0.5		0.7	0.9	0.6	0.5		0.7	0.6	0.6	0.6	
Alcohol consumption^b^, %					0.002					<0.0001					<0.0001
Abstainer and irregular consumer	54.7	51.5	51.4	54.6		51.7	50.8	51.9	57.7		61.1	55.0	49.4	46.6	
Moderate drinker	38.6	41.0	40.8	38.0		40.3	41.7	40.6	36.0		34.1	38.5	43.5	42.5	
Heavy drinker	6.7	7.5	7.8	7.4		8.0	7.5	7.5	6.3		4.8	6.5	7.0	10.9	
Smoking status, %					<0.0001					<0.0001					<0.0001
Never-smoker	51.3	52.5	53.1	52.8		47.8	51.2	53.3	57.3		56.0	54.1	52.6	47.0	
Former smoker	38.0	34.5	31.6	28.6		38.4	35.1	31.7	27.6		31.6	33.2	33.3	34.6	
Current smoker	10.7	13.0	15.3	18.6		13.8	13.7	15.0	15.1		12.4	12.7	14.1	18.4	
Dieting to lose weight, %					0.0003					<0.0001					<0.0001
Never dieter	17.9	16.9	16.3	16.7		16.2	15.7	16.7	19.1		19.2	17.5	15.8	15.1	
Former dieter	70.6	72.3	71.9	69.6		70.4	72.6	71.6	69.8		69.0	71.2	73.1	71.1	
Current dieter	11.5	10.8	11.8	13.7		13.4	11.7	11.7	11.1		11.8	11.3	11.1	13.8	
Physical activity, %					<0.0001					<0.0001					<0.0001
Low	15.6	22.6	26.0	31.4		19.8	21.8	24.5	29.6		21.5	21.8	24.8	27.5	
Moderate	41.8	44.8	47.0	44.0		42.9	44.4	45.8	44.4		43.5	45.7	44.8	43.5	
High	42.6	32.6	27.0	24.6		37.3	33.8	29.7	26.0		35.0	32.5	30.4	29.0	
Energy, kcal/day	1696.8 ± 397.4	1765.5 ± 397.3	1816.2 ± 414.7	1877.4 ± 448.8	<0.0001	1697.3 ± 397.3	1764.0 ± 409.5	1826.7 ± 417.6	1868.0 ± 436.2	<0.0001	1733.8 ± 412.3	1772.6 ± 411.3	1805.2 ± 411.5	1844.8 ± 437.6	<0.0001
Food group consumption, g/day															
Fruits	312.4 ± 191.1	276.3 ± 173.1	255.1 ± 167.0	226.0 ± 165.8	<0.0001	287.7 ± 191.2	272.6 ± 172.6	264.4 ± 173.1	245.1 ± 169.1	<0.0001	273.8 ± 185.7	270.8 ± 173.1	268.9 ± 174.6	256.3 ± 175.0	<0.0001
Meat	36.5 ± 38.7	40.1 ± 40.9	42.2 ± 40.5	44.5 ± 42.3	<0.0001	39.9 ± 41.3	39.6 ± 39.2	41.3 ± 41.0	42.5 ± 41.3	0.002	37.7 ± 39.9	40.1 ± 39.5	41.4 ± 40.1	44.0 ± 42.9	<0.0001
Processed meat	23.1 ± 26.8	27.8 ± 28.9	30.8 ± 30.8	33.2 ± 32.5	<0.0001	26.6 ± 29.4	28.1 ± 29.2	29.8 ± 30.8	30.6 ± 30.6	<0.0001	26.6 ± 29.6	27.5 ± 29.3	29.1 ± 28.9	31.8 ± 32.0	<0.0001
Milk and yogurts	165.7 ± 157.3	167.3 ± 151.3	168.6 ± 152.4	173.3 ± 159.2	0.10	168.8 ± 157.4	164.5 ± 149.7	166.1 ± 149.2	175.5 ± 163.4	0.004	175.1 ± 161.3	170.5 ± 155.3	167.3 ± 149.8	162.0 ± 153.3	0.0005
Cheese	31.1 ± 26.7	34.0 ± 27.7	35.6 ± 28.1	36.3 ± 29.1	<0.0001	32.7 ± 28.4	34.3 ± 27.5	34.9 ± 27.8	35.0 ± 28.1	0.0002	32.9 ± 27.6	34.3 ± 28.3	34.1 ± 27.1	35.6 ± 28.9	<0.0001
Oil	9.6 ± 10.0	9.0 ± 9.4	8.7 ± 8.8	8.7 ± 8.6	<0.0001	9.1 ± 9.4	9.1 ± 9.6	9.0 ± 9.0	8.8 ± 8.8	0.30	8.3 ± 9.2	8.8 ± 8.8	9.3 ± 9.3	9.5 ± 9.6	<0.0001
Whole grain products	45.3 ± 55.1	34.9 ± 45.3	30.5 ± 42.1	26.2 ± 40.7	<0.0001	40.8 ± 50.9	36.8 ± 48.4	32.0 ± 43.6	27.4 ± 42.3	<0.0001	38.9 ± 51.9	35.8 ± 48.3	33.0 ± 43.9	29.2 ± 41.4	<0.0001
Sugar and sugary products	22.4 ± 24.7	22.4 ± 23.2	21.8 ± 23.2	20.7 ± 23.0	0.001	15.6 ± 20.2	21.3 ± 23.2	23.5 ± 22.9	27.0 ± 26.1	<0.0001	20.1 ± 24.0	21.9 ± 22.4	23.1 ± 23.5	22.4 ± 24.1	<0.0001
Fatty-sweet products	48.9 ± 52.9	63.1 ± 56.2	73.3 ± 60.1	84.8 ± 68.2	<0.0001	53.3 ± 56.1	63.1 ± 58.1	72.18 ± 60.6	81.6 ± 65.4	<0.0001	66.9 ± 63.7	66.9 ± 60.6	66.1 ± 57.6	70.2 ± 62.1	0.006
Sugar-sweetened soft drinks	19.3 ± 56.4	28.2 ± 69.4	40.9 ± 95.2	54.6 ± 113.4	<0.0001	21.2 ± 66.8	26.1 ± 69.5	38.6 ± 88.1	57.0 ± 113.1	<0.0001	34.3 ± 84.0	33.4 ± 81.1	34.2 ± 81.4	40.9 ± 101.5	<0.0001
Artificially sweetened drinks	32.1 ± 136.8	36.0 ± 130.1	41.7 ± 134.3	47.0 ± 144.3	<0.0001	39.2 ± 154.3	36.6 ± 124.1	39.3 ± 133.7	41.6 ± 132.4	0.38	37.0 ± 135.3	37.8 ± 136.9	36.6 ± 131.0	45.8 ± 142.8	0.002

* *P* values are for the comparison between sex-specific quartiles of liking for fat, sweet and salt and were determined by using analysis of covariance or chi-square tests as appropriate

^a^ Mean ± SD (all such values)

^b^ Female drinkers were categorized as abstainer and irregular consumer (≤3 g alcohol/day), moderate consumer (>3 to ≤20 g alcohol/day), or heavy drinker (>20 g alcohol/day)

Lower intakes of fruit, oil, whole grain products, sugar and sugary products, and higher intakes of meat, processed meat, milk and yogurts and artificially sweetened drinks were significantly associated with obesity (Table 4).

**Table 4:** Baseline characteristics of subjects who became obese or not during the follow-up *n* = 24,776, NutriNet-Santé cohort, France

	Non-obese subjects	Obese subjects	
	*n* = 24,112	*n* = 664	*P* *
General characteristics			
Age, y ^a^	48.2 ± 14.2	49.8 ± 13.2	0.004
Sex, % women	75.1	75.8	0.68
BMI, kg/m^2^	22.7 ± 2.8	28.3 ± 1.3	<0.0001
Educational level, %			<0.0001
Elementary school	2.6	3.8	
Secondary school	32.6	42.9	
College graduate	30.1	30.3	
Advanced degree	34.0	22.1	
Other	0.7	0.9	
Alcohol consumption ^b^, %			0.03
Abstainer and irregular consumer	46.9	51.7	
Moderate drinker	44.1	39.0	
Heavy drinker	9.0	9.3	
Smoking status, %			0.15
Never-smoker	50.0	46.8	
Former smoker	35.9	39.5	
Current smoker	14.1	13.7	
Dieting to lose weight, %			<0.0001
Never dieter	21.3	3.6	
Former dieter	68.9	67.8	
Current dieter	9.9	28.6	
Physical activity, %			0.0001
Low	22.9	39.4	
Moderate	42.7	36.5	
High	34.5	34.2	
Sensory liking scores			
Liking for fat	3.79 ± 1.4	4.03 ± 1.4	<0.0001
Liking for sweet	3.73 ± 1.3	3.66 ± 1.3	0.12
Liking for salt	3.77 ± 1.5	3.93 ± 1.7	0.007
Food group consumption, g/day			
Fruits	278.7 ± 187.2	233.9 ± 187.8	<0.0001
Meat	44.8 ± 44.1	56.7 ± 52.8	<0.0001
Processed meat	31.1 ± 32.6	37.5 ± 40.8	<0.0001
Milk and yogurts	168.7 ± 156.7	192.4 ± 179.6	0.0001
Cheese	37.6 ± 30.8	35.9 ± 33.9	0.17
Oil	9.3 ± 9.6	8.1 ± 8.6	0.002
Whole grain products	36.0 ± 51.5	28.3 ± 43.3	0.0002
Sugar and sugary products	24.0 ± 25.5	18.9 ± 24.9	<0.0001
Fatty-sweet products	69.3 ± 63.1	65.0 ± 68.3	0.08
Sugar-sweetened soft drinks	38.8 ± 96.5	40.8 ± 97.9	0.60
Artificially sweetened drinks	35.6 ± 131.8	65.3 ± 165.1	<0.0001
Energy, kcal/day	1915.0 ± 501.1	1864.5 ± 548.2	0.01

* *P* values are for the comparison between obese and non-obese subjects and were determined using Student’s *t*-test or chi-square test as appropriate

^a^ Mean ± SD (all such values)

^b^ Male drinkers were categorized as abstainer and irregular consumer (≤4 g alcohol/day), moderate consumer (>4 to ≤30 g alcohol/day), or heavy drinker (>30 g alcohol/day) and female drinkers were categorized as abstainer and irregular consumer (≤3 g alcohol/day), moderate consumer (>3 to ≤20 g alcohol/d), or heavy drinker (>20 g alcohol/d)

Estimates of associations between sensory liking for fat and for sweet and the risk of obesity are presented in Table 5. In base models, higher liking for fat was associated with an increased risk of obesity (Men: HR_Q4vs.Q1_ = 2.39 (95 % CI 1.39,4.11), Women: HR_Q4vs.Q1_ = 2.02 (95 % CI 1.51,2.71)) compared to those with low fat liking (Table 5). Dietary factors explained 31 and 38 % of the decreased HRs in men and women in the highest liking category (quartile 4) respectively, and contribute to explain 32 and 52 % of the overall variation of fat liking in obesity, i.e. reduction in deviance of fat liking. In the fully adjusted model, the associations remained significant in men (P-trend = 0.01) and in women (P-trend = 0.015), and dietary factors and confounders explained together 36 and 72 % of the overall variation of fat liking, in men and women respectively. When we distinguished fat-and-salt and fat-and-sweet sensations in the overall fat sensation, we showed that liking for fat-and-salt was associated with the risk of obesity in men (base model: M: HR_Q4vs.Q1_ = 2.68 (95 % CI 1.50,4.81)) but not in women (P-trend = 0.11). In men, dietary factors explained 27 and 40 % of the decreased HRs in the quartiles 3 and 4 respectively, and contribute to explain 41 % of the overall variation of liking for fat-and-salt in obesity. Liking for fat-and-sweet was associated with an increased risk of obesity in women only (base model: W: HR_Q4vs.Q1_ = 1.68 (95 % CI 1.25,2.24)) and dietary factors explained 16 % of the decreased HR in the highest liking quartile and contribute to explain 22 % of the overall variation of liking for fat-and-sweet in obesity.

**Table 5:** Associations between quartiles of liking for fat, fat-and-salt and fat-and-sweet sensations, liking for sweet taste and natural sweetness and obesity risk from multivariate Cox proportional hazards model, *n* = 24,776

	Men *n* = 6175								Women *n* = 18,601							
	Quartile 2	Quartile 3	Quartile 4						Quartile 2	Quartile 3	Quartile 4					
	HR ^a^ (95 % CI)	HR (95 % CI)	HR (95 % CI)	P-trend	RHR1%^b^	RHR2%	RHR3%	RD%^c^	HR (95 % CI)	HR (95 % CI)	HR (95 % CI)	P-trend	RHR1%	RHR2%	RHR3%	RD%
Fat																
Base model	1.42 (0.85;2.37)	2.29 (1.29;3.77)	2.39 (1.39;4.11)	0.0005					1.29 (0.98;1.70)	1.41 (1.06;1.89)	2.02 (1.51;2.71)	<0.0001				
Model assessing the mediating effect ^d^	1.26 (0.75;2.10)	2.01 (1.21;3.32)	1.96 (1.13;3.41)	0.006	/	22	31	32	1.18 (0.90;1.56)	1.23 (0.92;1.65)	1.63 (1.21;2.20)	0.002	/	/	38	52
Fully adjusted model ^e^	1.24 (0.74;2.07)	1.99 (1.20;3.29)	1.85 (1.07;3.23)	0.01				36	1.20 (0.91;1.57)	1.22 (0.91;1.62)	1.49 (1.09;2.00)	0.015				72
Fat-and-salt																
Base model	1.71 (1.01;2.91)	2.40 (1.39;4.12)	2.68 (1.50;4.81)	0.0007					1.52 (1.16;2.00)	1.42 (1.06;1.91)	1.41 (1.02;1.95)	0.11				
Model assessing the mediating effect	1.45 (0.85;2.51)	2.02 (1.16;3.50)	2.13 (1.17;3.88)	0.008	/	27	40	41	1.39 (1.06;1.84)	1.25 (0.93;1.69)	1.16 (0.83;1.62)	0.76	/	/	/	34
Fully adjusted model	1.36 (0.79;2.33)	1.95 (1.12;3.40)	2.06 (1.13;3.78)	0.009				44	1.44 (1.09;1.90)	1.32 (0.98;1.79)	1.17 (0.84;1.64)	0.68				18
Fat-and-sweet																
Base model	1.06 (0.66;1.68)	0.93 (0.56;1.54)	1.14 (0.68;1.91)	0.69					1.04 (0.79;1.37)	1.29 (0.97;1.71)	1.68 (1.25;2.24)	<0.0001				
Model assessing the mediating effect	1.12 (0.70;1.78)	0.97 (0.58;1.60)	1.13 (0.67;1.91)	0.82	/	/	/	16	1.02 (0.77;1.35)	1.24 (0.93;1.64)	1.57 (1.18;2.11)	0.0006	/	/	16	22
Fully adjusted model	1.09 (0.68;1.74)	0.95 (0.57;1.57)	1.04 (0.61;1.76)	0.95				54	1.01 (0.76;1.33)	1.14 (0.86;1.52)	1.37 (1.02;1.84)	0.02				63
Sweet																
Base model	0.66 (0.42;1.03)	0.64 (0.40;1.01)	0.51 (0.31;0.83)	0.01					0.85 (0.66;1.09)	0.85 (0.65;1.10)	0.72 (0.54;0.96)	0.035				
Model assessing the mediating effect	0.69 (0.43;1.08)	0.74 (0.46;1.17)	0.56 (0.33;0.94)	0.056	/	/	10	31	0.94 (0.73;1.21)	0.96 (0.74;1.26)	0.86 (0.64;1.15)	0.38	/	/	/	76
Fully adjusted model	0.74 (0.47;1.16)	0.76 (0.47;1.21)	0.59 (0.35;0.99)	0.08				45	0.92 (0.71;1.18)	0.93 (0.71;1.21)	0.82 (0.61;1.10)	0.23				63
Natural sweetness component																
Base model	0.83 (0.55;1.23)	0.51 (0.32;0.81)	0.44 (0.27;0.71)	0.002					0.73 (0.57;0.92)	0.66 (0.52;0.84)	0.50 (0.39;0.65)	<0.0001				
Model assessing the mediating effect	0.94 (0.63;1.41)	0.66 (0.41;1.05)	0.63 (0.38;1.04)	0.15	/	/	/	66	0.82 (0.64;1.03)	0.80 (0.63;1.03)	0.66 (0.51;0.87)	0.03	/	/	32	68
Fully adjusted model	0.92 (0.62;1.39)	0.69 (0.43;1.10)	0.62 (0.37;1.04)	0.20				70	0.83 (0.66;1.06)	0.84 (0.66;1.08)	0.69 (0.53;0.91)	0.07				74

^a^ Reference category is quartile 1 “low liking”

^b^ % RHR: percentage reduction in HR by inclusion of mediator ((HR base model – HR base model + mediator)/(HR base model − 1))*100. RHR1 correspond to the reduction in HR of the quartile 2, RHR2 correspond to the reduction in HR of the quartile 3 and RHR3 correspond to the reduction in HR of the quartile 4. RHR was not calculated when none of the HRs were significant (/)

^c^ % RD: percentage of sensory liking reduction in deviance explained by inclusion of mediator and confounders ((reduction in deviance due to sensory liking of base model) − (reduction in deviance due to sensory liking of base model + mediator and confounders)/RD due to sensory liking of base model)*100

^d^ Model assessing the mediating effect: base model + food groups intake (fruits, meat, processed meat, milk and yogurts, cheese, oil, whole grain products, sugar and sugary products, fatty-sweet products, sugar-sweetened soft drinks and artificially sweetened drinks), energy intake and month of inclusion

^e^ Fully adjusted model: model assessing the mediating effect + educational level, alcohol consumption, smoking status, dieting to lose weight and physical activity

In base model, sweet liking was associated with decreased risk of obesity (M: HR_Q4vs.Q1_ = 0.51 (95 % CI 0.31,0.83), W: HR_Q4vs.Q1_ = 0.72 (95 % CI 0.54,0.96)) (Table 5). The associations were no longer significant when adding dietary factors which contribute to explain 31 and 76 % of the overall variation of liking for sweet in obesity, in men and women respectively. Detailed analyses in which we have considered the three factors composing the sweet taste: sweet foods, natural sweetness and added sugar were performed. Only liking for natural sweetness was significantly associated with the risk of obesity (base model: M: HR_Q4vs.Q1_ = 0.44 (95 % CI 0.27,0.71), W: HR_Q4vs.Q1_ = 0.50 (95 % CI 0.39,0.65)), but no longer significant when adding dietary intake and confounders. The other factors composing the sweet taste were not associated with the risk of obesity (liking for sweet foods, M: P-trend = 0.55; W: P-trend = 0.09 and liking for added sugar, M: P-trend = 0.44; W: P-trend = 0.34).

Regarding salt liking, a significant association with the risk of obesity was found in men only (base model: P-trend = 0.04) but the HRs were not statistically significant. Furthermore, this association was no longer significant when adding dietary factors in the model (data not tabulated).

In sensitivity analyses, results remained unchanged regarding sensory liking for fat, sweet and salt after including participants with missing data for confounders. Then, when the outcome was the risk of overweight, associations became non-significant regarding sweet liking and unchanged for fat and salt liking.

## Discussion

This prospective study reinforced results from cross-sectional studies, by highlighting that fat liking was prospectively associated with an increased risk of obesity and diet appeared to substantially explain this relationship. Results have also shown that sweet liking is associated with a decreased risk of obesity, and there is no significant association between salt liking and obesity risk.

Findings regarding the positive association between fat liking and the risk of obesity was concordant with most cross-sectional studies [5, 6, 9–14] and a longitudinal study [28]. Salbe et al. have shown a positive correlation between the hedonic response to sweet and creamy solutions and 5-year weight gain in the Pima Indians (*n* = 123) [28]. However, this same study has also been conducted in a group of white subjects (*n* = 64) and showed no significant association. Statistical analyses were Spearman correlations, so were not adjusted for well-known confounders such as physical activity, history of dieting or socioeconomic status, making the comparison with our results limited. The other available longitudinal study, performing statistical analyses adjusted for several confounding factors, has shown no association between the preference for “rich and heavy” taste and weight changes after 10 years [27]. However, the “rich and heavy” taste cannot be compared to the fat sensation as this referred to fat seasoning only and not to fatty-foods. In addition, only one question was used to assess sensory liking (“Do you like rich and heavy food” answer: dislike/neither/like), that does not allow comparing with our measure of liking for fat (liking for fatty foods, fat seasoning and eating behaviors regarding fat) which has been assessed by 51 items.

We have highlighted that dietary intake substantially explained the relationship between fat liking and the risk of obesity. Indeed, compared to those with low fat liking, subjects with high fat liking had an unhealthier diet [44], according to the French National Nutrition and Health Program, such as higher intake of total energy, meat, processed meat, cheese, fatty-sweet products, sugar-sweetened soft beverages, and lower intake of fruits, oils and whole grain products, concordant with a previous work which has studied the relationship between fat liking and dietary intake [4]. Individuals with high liking for fat may less consume nutrient-dense foods because they find them less tasty; consequently, they may tend to replace healthy foods by their energy-dense variants. Such dietary behaviors represent a nutritional difference between those with high fat liking and those with low liking (e.g. +200 kcal/day in the 4^th^ quartile of fat liking) that could have long-term consequences on weight gain and the risk of developing obesity.

Then, regarding components of fat sensation, a relationship between fat-and-salt liking and the risk of obesity was found in men only, whereas fat-and-sweet liking was associated with the risk of obesity in women only. These findings were in line with previous cross-sectional studies [5, 45] but no such distinction was found between genders. Regarding dietary intake, men with high fat-and-salt liking had also an unhealthier diet compared to those with low fat-and-salt liking and there was the same trend of an unhealthy diet in women with high liking for fat-and-sweet sensation. This specific gender association was concordant with a previous work which has shown higher intake of fatty-sweet products in women [53]. A review on food intake according to gender highlighted that consumption of sweet foods such as cakes, biscuits, puddings and chocolate is tacitly treated by men and women as a marker of femininity in many cultures, and a number of researchers note that meat products are commonly associated in everyday life with such qualities as strength, power and virility, a symbol of masculinity [54]. Our findings regarding fat-and-salt/fat-and-sweet liking and gender are in line with studies on fatty-salty/fatty-sweet foods intake and gender.

Our result showing that liking for sweet taste was inversely associated with the risk of obesity, and in particular liking for natural sweetness, are in line with a previous work of Cox et al. which has shown an association between “liking extremely” and “sweet foods” in lean subjects only, but no association in obese participants [20]. This result could be explained by the fact that individuals with high liking for natural sweetness (added jam, honey, ginger bread) had healthier dietary intake than those who have low liking for natural sweetness, such as higher intake of fruits (women (W): +27 g; men (M): +21 g) and whole grain products (W: +10 g; M: +12 g), and lower intake of meat (W: -6 g; M: -10 g), processed meat (W: -6 g; M: -4 g), sugar-sweetened soft drinks (W: -4 g; M: -4 g) and artificially sweetened drinks (W: -23 g; M: -8 g). However, our results are in contradiction with other studies [6, 19] including a longitudinal study [27]. Discrepancies between findings could be explained by the fact that studies reported in these citations were experimental [6, 19] and the longitudinal one used only one question “Do you like sweet food?” to assess sweet liking [27].

The differential associations of fat liking and sweet liking with the risk of obesity could be explained by several factors. First, subjects with high fat liking seemed to be less physically active and more often on diet than participants with high sweet liking, which could explain their higher risk of obesity. In addition, dietary intake and physical activity were assessed at baseline only. We cannot therefore evaluate the cumulative effects of these behaviors on the risk of obesity during the period of 5 years, that may be differential in subjects with high fat liking compared with those with high sweet liking. Finally, we could not adjust for the impact of other types of mediators of sensory liking in obesity such as genetics and psychological factors.

The non-significant association between salt liking and the risk of obesity over 5 years in our general population was concordant with most experimental studies [20, 22, 23]. Studies highlighting a significant association between salt liking and BMI were cross-sectional, used an unreliable measure of salt liking [24] or showed very small differences of salt liking scores between BMI categories [5].

Interpretation of the present results must take into account the characteristics of the study. Subjects were volunteers in the NutriNet-Santé cohort and probably more concerned about healthy lifestyle and nutrition than the general population. Caution is therefore needed when interpreting and generalizing the results. The prospective design of the study with the 5 years of follow-up allows us to explore the inference of causality between sensory liking and the risk of obesity. In contrast, individual characteristics and dietary intake were assessed at baseline only, so cumulative effect of these behaviors on the risk of obesity could not be assessed. Furthermore, as sensory liking has also been assessed at baseline, we did not have the evolution of liking over the 5-year period, which could vary. However, baseline sensory liking of excluded obese individuals and participants who became obese were compared and no significant difference has been found. Another potential limitation was that data were self-reported by questionnaire and could be not accurate as measured data. Indeed, compared with liking as assessed by sensory analysis, self-reported liking on a questionnaire may lead to misreporting. Recalled liking can be influenced by the recalled pleasure arising from the sensory cues, but also by other external cues such dietary habit, dietary restraint, social desirability, health considerations and other variables [33, 55]. However, this questionnaire was carefully developed through a series of pretests and pilots that demonstrated its repeatability, feasibility and internal validity [29], and positive correlations with sensory test measurements have been shown (Deglaire et al. 2011 personal communication). In addition, another study performed on a NutriNet-Santé cohort sample has demonstrated the validity of web-based self-reported anthropometric data by comparison with clinical data (*n* = 2513), and has shown that the reporting bias was reasonably small [56]. Finally, because our study investigated sensory liking with an epidemiological approach, some discordance between our results and the literature might be explained by differences in the methods employed. Indeed, we used a questionnaire, whereas other experimental studies generally used direct measures (e.g. solutions with salt or sugar).

## Conclusions

In conclusion, liking for fat sensation is a risk factor of obesity, whereas liking for natural sweetness is associated with a decreased risk of becoming obese. Our findings emphasize the need to consider the influence of sensory liking in the management and prevention of obesity. Taking into account an individual’s liking may help dietitians and practitioners provide effective dietary counseling while supporting individual liking. Further studies should be conducted to study relationships between sensory liking and other chronic diseases such as incident diabetes, hypertension or cardiovascular diseases.

## Abbreviations

BMI, body mass index; HR, hazard ratio; M, men; RD, reduction of deviance; RHR, reduction in hazard ratio; W, women.
